# The ethics of practicing defensive medicine in Jordan: a diagnostic study

**DOI:** 10.1186/s12910-021-00658-8

**Published:** 2021-07-07

**Authors:** Qosay A. E. Al-Balas, Hassan A. E. Al-Balas

**Affiliations:** 1 Department of Medicinal Chemistry and Pharmacognosy, Faculty of Pharmacy, Jordan University of Science and Technology, P.O. Box 3030, Irbid, 22110 Jordan; 2 Department of Radiology, Faculty of Medicine, Jordan University of Science and Technology, P.O. Box 3030, Irbid, 22110 Jordan; 3 Baylor College of Medicine, Houston, TX USA

**Keywords:** Defensive medicine, Litigation, Malpractice, Medical errors, Unethical practice

## Abstract

**Background:**

Defensive medicine (DM) practice refers to the ordering or prescription of unnecessary treatments or tests while avoiding risky procedures for critically ill patients with the aim to alleviate the physician’s legal responsibility and preserve reputation. Although DM practice is recognized, its dimensions are still uncertain. The subject has been highly investigated in developed countries, but unfortunately, many developing countries are unable to investigate it properly. DM has many serious ramifications, exemplified by the increase in treatment costs for patients and health systems, patients’ exposure to risks, and negative effects on the psychological health of both health providers and recipients. Ultimately, the most serious consequence is the ethical consequences.

**Methods:**

This work is based on a review of the literature related to DM worldwide and a comparison with the available knowledge found in Jordan. It is qualitative with a descriptive nature, aiming to diagnose the current DM practice in Jordan.

**Results:**

This is the first published article that discusses DM in Jordan by diagnosing its ethical and economic consequences for the health system as well as for patients. Despite the knowledge of the reasons that support its practice, little is being done to solve this issue. The absence of agreeable medical malpractice law, the dearth of unified medical protocols, the overwhelming pressure imposed by patients on medical staff, and the deteriorating patient-physician relationship are some of the causes of DM practice. Surely, the solution to these issues is to focus on fortifying the ethical and humanitarian aspects on the side of both the physician and the patient to ensure positive collaboration. The ethical aim of the physician to treat the patient faithfully and do what is possible to help combined with the appreciation of the physician’s efforts and the choice to not take advantage of the physician through litigation could be the most reasonable solution in the near future.

**Conclusion:**

Jordan is suffering from DM due to the limited financial expenditure on the health sector and the impracticality of medical malpractice law. The authors highlight that the cardinal step in solving this dilemma is restoring the ethical dimension of the patient-physician relationship.

## Introduction

In the modern era, humanity has never been more threatened by diseases than it has been during the current COVID-19 pandemic. All sectors in the field of medicine stand ready once again to combat its repercussions. Currently, humanity is highly dependent on medicine in response of the numerous diseases spreading worldwide [1]. Medicine has progressed in various stages and has been frequently castigated for being practiced unethically or by immoral means, with malpractice, medical errors, and defensive medicine (DM) as examples [2]. Defensive medicine as a term was coined in the 1950s and 60 s and can be interpreted as the physician’s aberration from proper medical practice with the aim to avoid criticism, litigation, and reputation sabotage [3, 4]. Although it was first recognized as a negative behavior adopted by physicians, others considered such behaviors favorable and legitimate [5]. Advocates of DM claim that it enables physicians to avert criticism, protect themselves from lawsuits, conserve their reputation and bypass complaints. Moreover, it may provide additional benefits for patients by helping discover previously unknown medical problems. In contrast, adversaries of DM claim that this behavior has detrimental ethical and professional consequences to the medical field. These consequences include the refusal to offer care to gravely infirm patients, the provision of unessential examinations or prescriptions, the exposure of other physicians to legal vulnerability, the incurrence of excessive and unnecessary costs and the poor example set for the education of future physicians [6].

Most of the cases of physician litigation at the beginning of the twentieth century stemmed from the accusation of erroneous behavior. Currently, the prosecution is veered to failure to perform the proper treatment for the patient [7]. Either way, is it ethically acceptable to believe the physician since he is the expert and to ignore the patient’s claim considering his predatory inclinations or, to the contrary, to believe the patient and accuse the physician?

## The dilemma of developing countries

A review of the literature indicates that few articles have investigated the impact of DM in developing countries. This can be attributed to many factors; for example, most developing countries suffer from immature or limited medical coverage for their citizens. Obviously, shortage or improper documentation in general and more specifically in the healthcare system is a hurdle for retrieving the data required for performing any analysis. The development of health care systems is highly reliant on external endowments that are the moral responsibility of developed countries, which are in many cases poorly managed and lack sustainability. Developing countries in general suffer imperfect strategic planning, usually with a short-term focus, and plans are frequently not executed given the many unexpected obstacles that arise [8].

For example, in Pakistan, one of the most populated countries in the world, more than 17.2% of the population lives in poverty, and only a small percentage enjoy full medical coverage, while the rest are dependent on out-of-pocket payment. Such a health system is incapable of bearing the consequences of DM practice, as the financial burden, ethical responsibility to the poor, and medical complications are inevitable [9, 10]. Several studies conducted in Turkey observed that, as in other countries, there was an increase in the practice of DM accompanied by an increase in litigations against doctors. Again, this increase led to more ethical, psychological and physical pressure on the medical systems and increased costs for both the patient and the insurance bodies [11, 12]. In 2017, Nepal enacted a health care reform, which is expected to cause a rise in DM practice, in response to fears of the inability to meet the overwhelming financial and human capital demand, with a potentially catastrophic effect on the country [13].

## Defensive medicine in Jordan

To the best of our knowledge, no study has considered the presence and scope of defensive medicine practice in Jordan. Accordingly, this investigational study screens the literature for publications that mention DM in general and related topics, such as medical malpractice, litigation against physicians due to medical errors and patient-doctor relationships. In this study, the authors will seek to understand the reasons that could trigger DM in Jordan, reveal how these reasons are reflected in everyday medical practice, and compare the case of Jordan with other countries in which such studies have already been carried out. Finally, suggestions and possible solutions will be formulated to help policy makers prevent such behavior and limit the negative impact on the country’s financial capital.

The world is currently a small village, and what happens in any part of it will affect other countries in one way or another. Jordan is not a separate country from the world, and DM practice in Jordan is likely to coevolve with the practice in the rest of the world for many reasons. First, many specialized physicians practicing in Jordan graduated from institutions in the USA and European countries after obtaining their first degree in medicine and being sent by the government or going on their own to pursue subspecialization in renowned universities. When they returned to Jordan, they implemented what they learned in their daily practice. DM is part of what they acquired and implemented, even if they may not fully perceive their engagement in this practice. Another reason could be the increase in litigation against physicians in recent years and the recent approval of malpractice law in Jordan in 2018. Globalization and the strength of Western culture have affected the values, morals and ethical grounds of both physicians and medical service recipients [14, 15].

### Medical practice in Jordan

The health system organization in Jordan comprises three main sectors: the public health system, which is the largest, the private sector and the donor sector. The public health system is subdivided into the Ministry of Health (MoH), university hospitals, and Royal Medical Services (RMS). Most Jordanians are insured by one of these sectors, and the bulk of the burden is on the MoH and RMS, which together cover two-thirds of the insured [16]. Among the Arab countries, Jordan has been classified as number one in offering medical tourism and is one of the top ten countries in this strategic field. Consequently, Jordan makes efforts to preserve this status quo and even to fortify this trend by attracting patients from nonconventional markets. General factors that have allowed Jordan to achieve this position include the presence of highly qualified medical personnel, the quality medical services available at competitive prices, a large number of internationally accredited hospitals and political stability and security [17].

On the other hand, the medical sector is also suffering from many drawbacks including the weak implementation of cost containment strategies, the duplication of the health insurance system, increasing out-of-pocket spending, the unplanned and impromptu expansion of medical services, the unethical practices of some physicians, and a steady upsurge in health care costs accompanied by a lack of funding [17].

### Malpractice in Jordan

Malpractice is defined by the Merriam-Wester dictionary as “a dereliction of professional duty or a failure to exercise an ordinary degree of professional skill or learning by one (such as a physician) rendering professional services which results in injury, loss, or damage” (Merriam-webster 2021). Malpractice has been present since medicine first started to be practiced and is not confined to developing countries; rather, it is found in all health systems worldwide, including well-developed countries with advanced health care systems. In Jordan, malpractice is likewise present. Unfortunately, detailed statistics are not available to measure the scope of the problem, which is related to many factors. Litigation of physicians who commit malpractice is possible but is not straightforward. Patients are allowed to sue their physicians in the courts, but this process is tedious and cumbersome and in most cases results in abandonment of the litigation process. The litigation process is estimated to take 534 days, on average, with a small chance of obtaining compensation. Moreover, if compensation is awarded, it is likely to be a small amount to discourage patients from pursuing malpractice issues [18]. A comprehensive study was conducted in 2007 with the help of the USAID entitled “MEDICAL MALPRACTICE LAW ‘BEST PRACTICES’ FOR JORDAN”, which recommended that Jordan adopt a suitable law for medical malpractice. Once enacted, this law will have potentially profound benefits for medical tourism and thus attract patients from nonconventional markets as well as patients from new and important markets, such as Europe and North America. Additionally, the patients from the conventional markets will have high trust in the medical system, which will encourage other patients to seek medical treatments [19]. Unfortunately, the medical malpractice law has recently been approved but is still not effectively applied in practice due to many logistic factors, most importantly the COVID-19 crisis. Moreover, there are many ethical, legal and logistic issues related to the best way to implement the law that are subjects of contestation between the MoH and the physicians, represented by the Jordanian Medical Association (JMA), and many issues are still unsolved. One of the main issues is the fact that public sector hospitals are overwhelmed by patients, and doctors in this sector have to handle and treat many more patients than their counterparts in other countries. Handling more than the recommended number of patients makes mistakes more likely since physicians are exhausted and less focused. A strategic and comprehensive plan should be implemented to enact a reasonable and applicable law that assuages the existing tension between patients’ rights and doctors’ integrity.

### The patient–doctor relationship

The Hippocratic oath, which was enacted in the fourth or fifth century BC, has lingered as the reference for medical practice worldwide. It began as an implicit ethical contract between the patient and the physician and has persisted as an indicator of a healthy relation until recently. The unprecedented scientific advancement in all aspects of life in general and conspicuously in medicine has changed this understanding between the two parties. Patients have high expectations concerning the treatment quality, recovery and low complications and mortality rates, and when these are not fully achieved, violent repercussions against medical staff can result. As expected, physicians seek refuge in defensive medicine to alleviate the pressure, protect their integrity and guarantee their continued practice [20].

In Jordan, although the patient-physician relationship observable worldwide has led to a noticeable shift, the paternalistic relation still predominates. This culture is augmented by patients’ respect and admiration of physicians and their trust that the physician will make the proper decision. Moreover, the Muslim community believes that their circumstances are subject to “fate and destiny”, and they have to accept what happens even if not all proper medical practice criteria are followed. New generations of patients and medical tourists expect more from medical staff, resulting in a shift to more customer-based relations. Globally, more aggressive acts have been documented against physicians in the form of litigation and disrespect, making physicians seek diverse protective strategies, including DM [21].

## Ethical reflections

It is onerous to accept the assumption that physicians intentionally commit harm to patients. Worldwide, physicians’ qualification process is generally robust, and candidates are subjected to a series of filtering and formulative stages both scientifically and ethically, as they are bound to code ethics. The most likely justification of the sensitive relation between the service recipient and provider is unintentional errors or occasionally ignorance. These scenarios could be managed by balancing the rights of the medical service recipients and the physicians’ obligations by formulating and enacting a medical law that satisfies all the parties involved in the process. Such a law would ensure that both Jordanians and patients in Jordan for medical tourism receive compensation in case of any malpractice. The New Zealand model could be a suitable option, in which compensation for patients in case of preventable trauma is offered by the government, while the litigation or suing process of physicians is executed by their professional body if they are proven guilty [6].

## Consequences of practicing defensive medicine in Jordan

### Health consequences

As a natural retreat from the growing pressure on physicians, critically ill patients can be declined treatment in some hospitals and transferred to other hospitals under claims of a shortage of specialized staff and instrumentation. This increases the risk of mortality while the patients are being transported from one hospital to another. When patients are hospitalized, ordering unnecessary examinations and tests in conjunction with unessential surgeries could compromise the health status of the patients due to the surgical risk in addition to increased chances of nosocomial infections. For example, ordering a CT scan is considered a paramount health risk to the patient due to the high dose of radiation that exposes him to real danger of overradiation [6]. Emergency Medical Treatment and Labor Act “EMTALA” like law application in Jordan could protect patients from being discharged or mobilized to other hospitals unless this is proven to be inevitable. All medical services should be provided to the patients within the capacity of the emergency department in that hospital. This will lessen the danger that could happen to the patients from transfer or refusal for treating them based on their complicated medical condition [21].

### Financial consequences

Although related research in Jordan is scarce or even nonexistent, the financial costs do not differ from those in other countries, either developed or developing. The hidden cost of defensive medicine is difficult to discern, although its existence can be appreciated. Scores of articles have investigated DM in developed and developing countries, and all have highlighted its high costs; therefore, its presence in Jordan is very likely [3, 9–11, 23, 24]. It is estimated that the DM forfeiture was US$12 billion in the United States in 1987 and double that in the next decade [25]. Moreover, losses due to DM account for approximately 5–9% of the total health budget in the US [5]. While this causes indisposition (ethically and professionally) in the US despite its high health care budget compared with the budgets of other countries, it is even more logical for countries with tight budgets to monitor such behavior and enact regulations to limit this practice. Jordan, as a developing country with a limited health budget and minimal medical services offered to its citizens, should be highly committed to identifying the areas of unnecessary expenditure and investing in infrastructure that could help divert waste money to more useful medical services.

The eradication of DM behavior is challenging, with many advanced countries struggling to limit its practice and costs. Similarly, Jordan should adopt strategies and action plans as soon as possible to control this behavior before it is more broadly disseminated and jeopardizes the already compromised health care system. The absence of nationally binding treatment protocols along with inefficient and partially implemented malpractice law has contributed to the emergence of DM practices.

### Professional consequences

The reputation of the physician is considered his capital; therefore, he claims that he has the right to take suitable actions (sometimes ethically questionable) to defend his reputation and his sole source of income. The approval of malpractice law in Jordan in 2018, the increase in litigation cases against physicians, the rise of aggressive actions against medical staff, the increase in patients’ awareness of their rights, advanced training in the US and Europe, the absence of career insurance for doctors, and the changing nature of the physician–patient relationship to a customer focus have all contributed directly or indirectly to the emergence of defensive medicine behavior. However, it is difficult to outline boundaries to discriminate between DM and proper practice. This is because practicing medicine is partially based on judgments and the context of the case, with vast gray area [26]. Physicians can employ the “adaptive prey” model to keep pace with their profession and balance risks and benefits [5]. Active prey model is a predator–prey relationship practiced between the patients and the physicians. Litigious patients are considered the predators as they seek compensation while the physicians are modifying their behavior by using DM.

### Psychological consequences

Psychological effects have a preeminent status for both physicians and patients. The Jordanian community shows high sympathy to the weak or the sufferer. Therefore, it is intolerable to witness a patient’s inadequate treatment or medical failure out of physician’s fear of medical responsibility or relatives’ aggressive actions. Such defensive medicine behaviors preclude any efforts to rebuild patient-physician relationships and restore the already deteriorated trust in the medical system.

In contrast, the psychological status of the physician is critical to the proper delivery of proper treatment, and a healthy status can be achieved only by providing a comfortable environment free of threats against the physician. There should be a harmonized formula capable of balancing the duties of physicians and the rights of patients in an agreeable medical decree. It is unimaginable that a practitioner, if he makes an error, could be sued and his career ruined. The overwhelming insecurity caused by this possibility would make him more prone to make errors. However, an excessive sense of security and freedom should also not be granted, and physicians should remain under a regulatory umbrella that entails the right to question negligence and misconduct and protect patient rights [27].

### Education quality

Fear of litigation is the major contributor to the flourishing of defensive medicine. Once practiced by senior physicians, it will be disseminated unconsciously and unintentionally to trainees or other senior doctors. Then, this practice will become an integral part of the treatment process or protocol, as medicine is based on experience and skill transfer processes rather than self-study. In Jordan, the last three years of the bachelor’s degree in medicine involve practice in clinics and hospitals, by which the professional experience of practicing doctors is transferred to medical students. In addition, residency programs, as internationally agreed upon, are based on a mentorship process, in which the senior physician passes his expertise to his fellow. Eventually, this knowledge becomes tacit knowledge for the trainee or the junior doctor and is, in turn, passed on to next generations. This snowball of defensive medicine, if not managed wisely, will cause substantial ethical, psychological, educational and financial issues, with unpraiseworthy consequences [10, 28, 29].

### Advisable solutions

While the most recent medical act in Jordan was established in 2018, there are many obstacles to its proper application, especially in the public sector. The overwhelmed medical sector with a scarcity of specialized physicians is the main obstacle. Many specialized physicians work in various MoH and military hospitals to alleviate the shortages of medical staff in peripheral hospitals. On the other hand, the private sector attracts highly skilled doctors with high salaries and small workloads. Importantly, the neighboring wealthy gulf countries also attract eminent consultants to serve in their health systems given their better working environment and less stressful climate, as reported by the Jordanian Medical Association [29].

The political instability in surrounding countries, such as Syria, Yemen, Palestine and Iraq, has been considered a main issue compromising the efficiency of health services due to the influx of millions of refugees since 1948. The collective ethical and humanitarian duty of both the government and the people in Jordan has imposed the ethical need to help the needy, with excellent examples of altruism regarding medical help and many other aspects. The help of international organizations and countries is gratefully acknowledged, but the colossal burden is on Jordanian institutions [30]

Solving the conundrum of defensive medicine is a laborious task for policy makers worldwide, but it is more challenging in Jordan due to the complex factors mentioned above. One of the most important criteria of the authors is the ethical dimension of the problem, which is exemplified by the restoration of the decayed patient-physician relationship. On the patient side, it is important to expect the sincerity, professionalism, and good disposition of the physician, while on the other side, the physician should ethically and legally conform to his expected role of employing his knowledge and efforts to his patient’s benefit, even sometimes at the expense of his own time and comfort. Philanthropy is a sacred value for Muslims and is deeply rooted within Jordanian community culture. Thus, it is important to successfully employ and disseminate this value to revive the deteriorating patient-physician relationship, emphasizing that no physician would intentionally harm his patient. At the same time, the physician should consider the patient his cognate and make his best efforts to treat the patient and alleviate his suffering [31].

It has been reported that physicians in general overestimate their legal risks, increasing the practice of DM in the US and leading a large percentage of surgeons in the US to view every patient as a looming lawsuit [32]. The enactment of the current liability law in Jordan is expected to lead the same trend to prevail in Jordan, with a key difference: the Jordanian financial health system is in no position to be compared to the US system. Therefore, any money lost to defensive medicine in Jordan will have a considerable impact on the overall health system, and the effect of the waste of a given amount of money will be felt much more in Jordan than in developed countries. Jordan cannot afford the practice of defensive medicine.

A professional juridical system with adroit jurists and experts who can protect physicians from forgery or false accusations is one decisive way to make physicians feel protected. Moreover, the Jordanian Medical Association (JMA) could represent a protecting and monitoring lobbying power. On the other hand, the rights of the patients must be guaranteed and nurtured, remembering that his human dignity and integrity represent the capital of his existence. Any of us could be a patient one day, and we all require proper treatment and management.

## Concluding remarks

DM is an international dilemma that affects both developed and developing countries. The core reason for its existence is ethical in principle. However, there are many other reasons, including fear of litigation and loss of reputation, that lead physicians to practice DM. Jordan is not immune to such behavior, which has exacerbated effects due to the limited financial expenditure on the health sector and the unsuitability of medical malpractice law. The authors highlighted that the cardinal step in solving this dilemma is restoring the ethical dimension of the patient-physician relationship; other solutions can be achievable only once the first step is accomplished.

## Data Availability

The data are available upon request from the corresponding author qabalas@just.edu.jo.
